# Quantitative Succinylacetone Measurement by Gas Chromatography‐Tandem Mass Spectrometry (GC–MS/MS) Facilitates Diagnosis, Monitoring, and Characterization of Tyrosinemia Type 1 and Other Hypersuccinylacetonemias

**DOI:** 10.1002/jmd2.70078

**Published:** 2026-02-27

**Authors:** Denis Cyr, Bruno Maranda, Paula J. Waters

**Affiliations:** ^1^ Medical Genetics Service, Department of Laboratory Medicine University of Sherbrooke Hospital Centre (CHUS) Sherbrooke Quebec Canada; ^2^ Department of Pediatrics University of Sherbrooke Sherbrooke Quebec Canada

**Keywords:** gas chromatography‐tandem mass spectrometry, hypersuccinylacetonemia, maleylacetoacetate isomerase, succinylacetone, tyrosinemia

## Abstract

Tyrosinemia type 1 (HT1), due to deficient activity of fumarylacetoacetate hydrolase, causes accumulation of succinylacetone (SA). SA concentrations in urine and plasma of untreated HT1 patients are typically several thousand‐fold higher than normal, hence are readily recognized by traditional diagnostic methods in most cases. However, quantitation of SA in the nanomolar range is important for monitoring patients treated with nitisinone, for identifying attenuated or atypical forms of HT1, and for confirmation or refutation of the diagnosis of HT1 following a positive newborn screen. Our laboratory, a reference centre for diagnosis and monitoring of HT1, previously assayed SA by gas chromatography–mass spectrometry (GC–MS). Three years ago, we upgraded this method by transferring it to a new triple quadrupole technology (GC–MS/MS). A stable isotope dilution process is used, with sample treatment consisting of an oximation step followed by a single liquid–liquid extraction then trimethylsilyl derivatization. Quantitation is based on intensities of the ion transitions m/z 620 → 181 for SA and 625 → 186 for the internal standard. Method validation demonstrated enhanced analytical specificity and sensitivity, with good precision and accuracy. Using GC–MS/MS instead of GC–MS allowed a limit of quantitation of 1 nmol/L while decreasing the required specimen volumes, as well as reducing the number of sample processing steps, chromatographic run time, and instrument maintenance. This assay facilitates laboratory diagnosis and monitoring of HT1, permits identification and characterization of other hypersuccinylacetonemias including maleylacetoacetate isomerase deficiency, and is also a valuable tool for research studies using animal models and cellular models of HT1.

## Key Points

- A novel method for succinylacetone quantitation, using gas chromatography–tandem mass spectrometry, was developed and validated.
- The main advantages of this method, compared to other existing succinylacetone assay methods, are enhanced sensitivity and specificity.
- Data from three years' routine application in a clinical laboratory demonstrates the method's utility in diagnosis and monitoring of tyrosinemia type 1.

## 1 Introduction

Tyrosinemia type 1 (HT1; OMIM #276700) is an autosomal recessive disorder of amino acid metabolism, characterized by severe liver disease, renal tubular dysfunction and neurological sequelae if the disease is not treated. It is caused by pathogenic variants in the *FAH* gene, which encodes fumarylacetoacetate hydrolase (FAH; EC 3.7.1.2), the final enzyme in the pathway of tyrosine catabolism [1, 2]. Loss of FAH enzymatic activity leads to accumulation of the toxic metabolites fumarylacetoacetate and succinylacetoacetate. Succinylacetoacetate is readily decarboxylated to succinylacetone (SA), which is the primary biomarker used for screening and diagnosis of HT1 [1, 2, 3, 4, 5].

The worldwide incidence of the disease is estimated at approximately 1:100000 [6] but HT1 shows higher incidence in certain populations due to founder effects [2, 7]. In the province of Quebec, in Canada, an unusually high incidence prompted introduction of the world's oldest and longest‐running program for HT1 newborn screening (NBS) [7, 8]. Nitisinone (NTBC, 2‐(2‐nitro‐4‐trifluoromethylbenzoyl)‐1,3‐cyclohexanedione), a potent inhibitor of 4‐hydrophenylpyruvate dioxygenase [9], reduces the production of toxic metabolites in HT1. Treatment with NTBC, combined with dietary tyrosine restriction, dramatically improves the clinical course [10] and has become the mainstay for the management of patients with HT1 [1, 4, 5].

Quantification of SA in the nanomolar range, in urine, plasma and/or dried bloodspots, is recommended for optimal monitoring of treated patients [4, 5, 11, 12]. A sensitive, specific assay for SA also has an important role in the confirmation or refutation of diagnosis following a positive newborn screen for HT1 [4, 5]. In 2006, our laboratory established a sensitive SA assay [13] using gas chromatography–mass spectrometry (GC–MS) for testing of urine, plasma and amniotic fluid samples. This enabled the laboratory to serve as a reference centre for SA analysis for Quebec and several other Canadian provinces, and also led to the identification of genetic causes of hypersuccinylacetonemias other than HT1 [14, 15].

Here we describe a significant upgrade of the previous assay, now using a gas chromatograph interfaced to a triple quadrupole mass spectrometer (gas chromatography‐tandem mass spectrometry; GC–MS/MS). By selecting precursor–fragment ion transitions specific for SA and ^13^C_5_‐SA (internal standard), we were able to improve the sensitivity and specificity while also streamlining the analysis to facilitate its routine use in the clinical laboratory. Three years after implementation, this current report presents a new validated isotope dilution method for measuring SA. Data are provided to illustrate its various applications in diagnosis and monitoring of HT1 and its utility for research related to disorders of tyrosine metabolism.

## 2 Material and Methods

### 2.1 Chemicals and Reagents

Succinylacetone (4–6‐dioxoheptanoic acid) and O‐(2,3,4,5,6‐pentafluorobenzyl)hydroxylamine hydrochloride (PFBHA) were obtained from Sigma (St. Louis, MO, USA); ^13^C_5_‐ succinylacetone (^13^C_5_‐SA stable isotope internal standard) from Cambridge Isotope Laboratories (Cambridge, MA, USA). In all cases, we used the highest grade commercially available. *N*,*O*bis‐trimethylsilyl‐trifluoroacetamide (BSTFA) + 10% Trimethylchlorosilane (TMCS) was from Regis Technologies (Morton Grove, IL, USA), and ACS grade ethyl acetate from Fisher Scientific (Ottawa, Canada). A stock solution of SA (200 nM), corresponding to the highest point of the calibration curve, was prepared in water, aliquoted and stored at −80°C. A stock solution of ^13^C_5_‐SA (30 mM), the stable isotope‐labelled internal standard, was prepared in water and stored at −80°C. From this solution, a working solution (2 μM) was made in water, aliquoted and stored at −80°C.

### 2.2 Sample Extraction and Derivatization

A 5‐point calibration, ranging from 12 to 200 nM, was prepared daily by appropriate dilution of the stock solution. To 1 mL of each standard, urinary quality control specimen, patient urine sample (with a creatinine concentration adjusted with water to approximately 1 mM) and amniotic fluid sample, or to 0.5 mL of each plasma sample and plasma quality control specimen (brought up to 1 mL with deionized water), was added 50 μL of ^13^C_5_‐SA (2 μM) internal standard plus 40 μL H_2_SO_4_ 0.25 M and 100 μL PFBHA (50 mg/mL) in order to perform oximation for 60 min at room temperature [16]. Oximation stabilises the oxo form over the enol form by producing oximes which are chemically more stable [17]. The pH was adjusted to 1 by adding 40 μL 5 M HCl to each tube, followed by saturation with 0.1 g sodium chloride. The sample was then extracted once with 2 mL ethyl acetate, with vigorous shaking. The organic layer was transferred into a second tube and the solvent was evaporated to dryness under a gentle stream of nitrogen. The residue was then derivatized by adding 100 μL BSTFA +10% TMCS and heating for 60 min at 70°C. One microliter of each sample was injected into the GC–MS/MS system. Urinary creatinine concentration is determined separately by an enzymatic method in our core clinical biochemistry laboratory, using a Roche Cobas Pro unit.

### 2.3 Gas Chromatography and Tandem Mass Spectrometry Conditions

Method improvement was performed using an Agilent 7010B Triple Quadrupole GC–MS/MS equipped with a high‐efficiency electron impact ionization source (HES). Chromatographic separation was achieved with two Phenomenex Zebron ZB‐5MSi columns (length, 15 m; internal diameter, 0.25 mm; film thickness, 0.25 μm) installed in tandem. This set‐up allows a backflush of the first column during the run, avoiding contamination of the second column and of the detector. The helium carrier‐gas flow rate was 1.2 mL/min in column 1 and 1.0 mL/min in column 2. The GC temperature program was as follows: initial temperature was 120°C, held for 1 min, then increased to 290°C at a rate of 22°C/min and held for 2.5 min. Finally, the oven temperature was increased to 300°C at a rate of 40°C/min and held for 5 min; resulting in a total run time of 15.5 min. A split injection mode (Split ratio = 5:1) was used and 1 μL was injected at 250°C. Transfer line temperature was 280°C and ion source temperature was 230°C. In Multiple Reaction Monitoring (MRM) mode, nitrogen and helium were used as collision gas and quench gas respectively. MRM is used for the MS recording of the ion transitions, *m/z* 620 → 181 for SA (qualifier *m/z* 620 → 225), and for the internal standard, ^13^C_5_‐SA *m/z* 625 → 186 (qualifier *m/z* 625 → 230).

## 3 Results

During the first stage of method development, SA and ^13^C_5_‐SA were analysed separately in scan mode and the tandem mass spectrometry parameters were refined using the MassHunter Optimizer for GC/TQ (https://www.agilent.com). This software identified precursor ions and fragment ions, optimized collision energies and found the best MRM parameters. Further experiments were also conducted using different mass spectrometric parameters (collision cell energy, mass resolution, gain, dwell time and source temperature) to find the most specific ion transitions.

**FIGURE 1:**
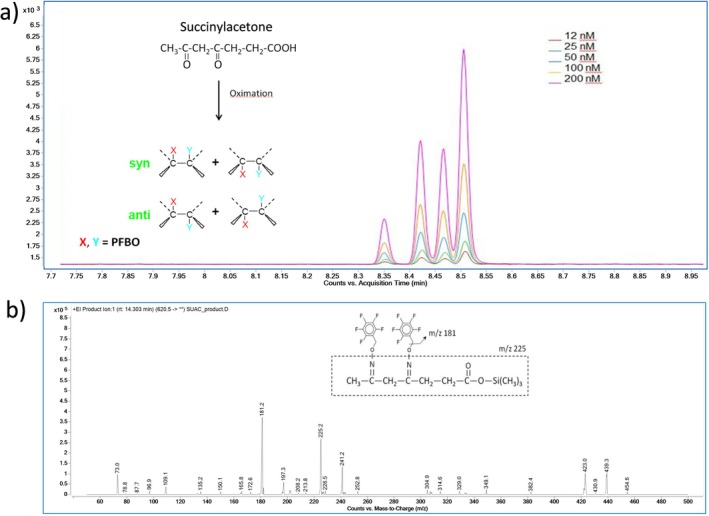
(a) Chromatogram of succinylacetone dioxime‐trimethylsilyl derivatives, showing the characteristic pattern of four peaks, each representing one of four different isomers. (b) Mass spectral fragmentation of the precursor ion *m/z* 620.

Figure 1 shows the chromatographic separation and the fragment ion mass spectra and molecular structures of SA dioxime‐trimethylsilyl (TMS) derivatives obtained using this GC–MS/MS method. SA was converted into four different dioxime derivatives when treated with PFBHA. After trimethylsilylation, the four isomers, comprising two *cis* and two *trans* chemical configurations, were separated by capillary GC, producing a distinctive chromatographic pattern. This characteristic motif of four peaks, combined with tandem mass spectrometry targeting the specific ion transitions *m/z* 620 → 181 and *m/z* 620 → 225 (qualifier), greatly enhances the method's specificity by permitting unambiguous identification of SA. The pattern is reproducible, i.e., the relative abundances (ratios of areas) of the four peaks are consistent across different specimens or calibration standards, irrespective of the SA concentration. Similarly, the internal standard, ^13^C_5_‐SA, also yields a corresponding, reproducible chromatographic pattern, and ensures specificity when the respective ion transitions *m/z* 625 → 186 and *m/z* 625 → 230 (qualifier) are targeted by tandem mass spectrometry (not shown). In order to establish the standard curve and perform sample quantifications, the ion abundance of the most prominent isomer was routinely used. This approach, rather than needing to calculate the sum of the abundances of all four peaks in the sample and the sum of the abundances of the corresponding four peaks in the internal standard, allows simpler and more rapid data analysis, while achieving the same degree of accuracy and sensitivity.

**FIGURE 2:**
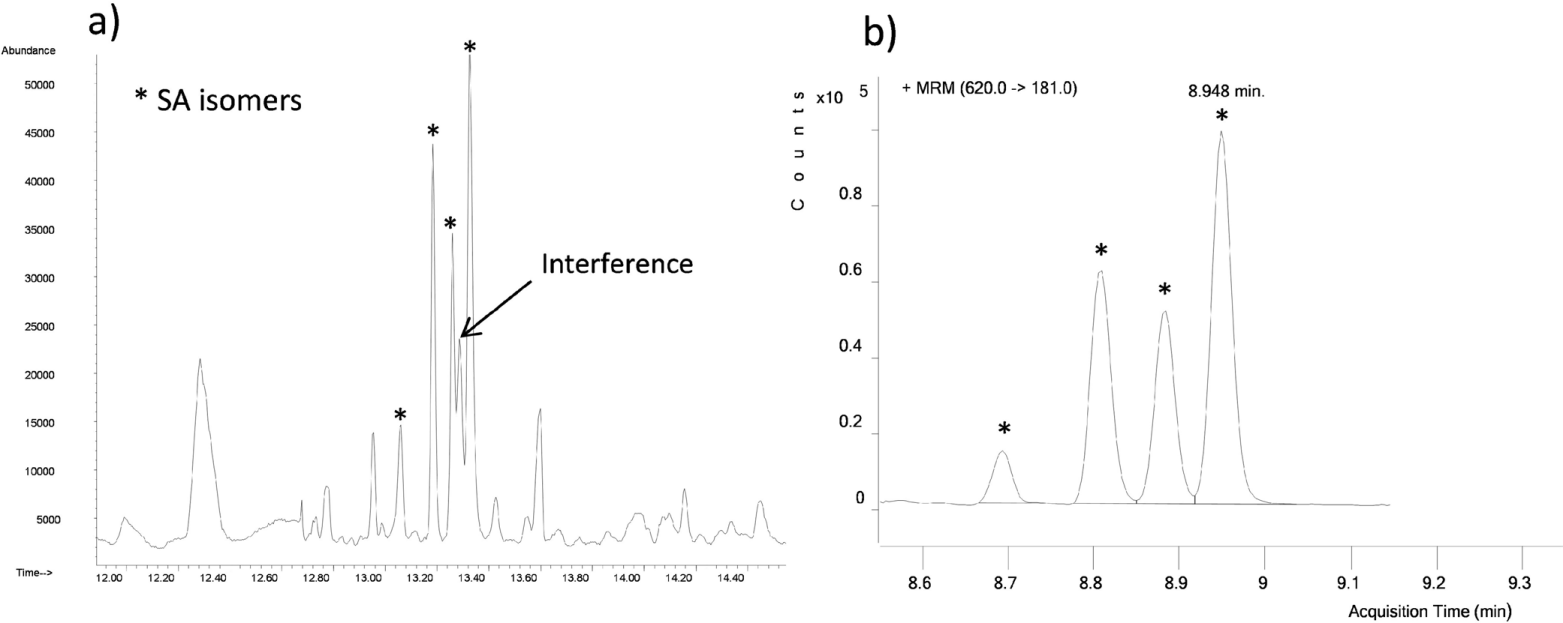
Analytical specificity: Comparison of previous GC–MS method versus current GC–MS/MS method. (a) The left‐hand graphic shows the chromatographic trace obtained from GC–MS analysis of a certain urine sample containing an interfering substance. (b) The right‐hand graphic shows the trace obtained by GC–MS/MS analysis of the same urine sample. Succinylacetone concentration was 95 μmol/mol creatinine.

The benefits of this method are clearly illustrated in Figure 2. The chromatogram obtained from GC–MS/MS analysis is free of the interferences caused by co‐eluting peaks that are commonly problematic in GC–MS and shows a clean baseline with minimal “noise”.

The method was validated according to standard criteria, based on ISO15189 (www.iso.org) and COFRAC guidelines [18]. Results are summarized in Table 1. The evaluated parameters included limit of quantification (LOQ), linearity, measurement range, precision, accuracy and recovery. Successive dilutions of normal urine and plasma spiked with SA were analysed and the lowest calculated concentrations with a relative error < 20% were used to establish the lower limits of quantification (LOQ). The upper limits of the measurement ranges were evaluated using samples with high SA concentrations obtained from the ERNDIM (European Research Network for evaluation and improvement of screening, Diagnosis and treatment of Inherited disorders of Metabolism; www.erndim.org) external quality assurance schemes Special Assays in Urine (SAU) and Special Assays in Serum (SAS), with the mean of results from all participants used as the target value and relative error < 20% considered acceptable. The linear regression of the calibration curve, using a concentration range between 0 and 200 nmol/L, showed an r^2^ close to 1. Intra‐assay precision was assessed with triplicate data from 3 levels obtained during the LOQ experiment. Internal quality control samples in urine and plasma matrices at low and medium/high levels were used to analyze the inter‐day precisions. While the inter‐assay CVs for the low controls were above 20%, we note that those controls were pooled urine and plasma from healthy adults, without addition of succinylacetone, in which the endogenous SA concentrations were close to the LOQ. These low controls adequately fulfilled their primary purpose, to ensure that they consistently showed results well within reference range.

**TABLE 1:** Summary of method validation results.

Criteria	Succinylacetone (SA) by GC–MS/MS	
	Urinary SA	Plasma SA
Limit of quantification (LOQ)	1 nmol/L	1 nmol/L
Linearity (r^2^) ‐ 0 to 200 nmol/L	0.999	0.999
Measurement range limits	1–1800 nmol/L^a^	1–3600 nmol/L^a^
Precision (intra‐batch)		
Low value	0.8%	2.2%
Medium value	3.7%	0.8%
High value	2.4%	2.9%
Precision (inter‐day)		
Low value	23,2%^b^	37,9%^b^
High value	6.7%	10.6%
Accuracy	93.0%	99.5%
Recovery		
Low value	84.4%	83.6%
Medium value	89.5%	91.1%
High value	81.5%	92.1%
Reference range	< 34 μmol/mol creatinine	< 24 nmol/L

^a^ Highest tested concentration.

^b^ Normal concentration, near the LOQ.

Accuracy was evaluated by participation in ERNDIM's SAU and SAS quantitative external quality schemes, respectively including eight urine and eight serum samples per year, spiked at four different concentrations covering a wide range of physiological and pathological levels. The overall accuracy results presented in Table 1 are compared to the mean results of all participating laboratories (https://www.erndim.org/eqa‐schemes/eqa‐scheme‐annual‐reports/). The recovery of SA was initially evaluated by analyzing plasma and urine samples spiked in our laboratory with known concentrations of SA at three different levels. Performance for accuracy, as well as for recovery, linearity, and inter‐day precision, has also remained satisfactory according to ERNDIM's criteria throughout our three years' participation using this GC–MS/MS method (data not shown). Finally, carryover (potential for inter‐sample contamination) of SA was assessed at 0.1%.

The diagnostic performance of the GC–MS/MS assay was further validated by prospective analysis of urine and plasma samples in regular clinical practice. Table 2 presents urine and plasma SA results observed at the time of diagnosis of HT1 or of hypersuccinylacetonemia due to maleylacetoacetate isomerase (MAAI) deficiency; in each case subsequently confirmed by molecular analysis of the *FAH* or *GSTZ1* genes, respectively. In untreated HT1 patients, SA concentrations in both urine and plasma were several thousand times higher than the normal (nanomolar) levels. While specimens from individuals with MAAI deficiency exhibited elevated SA concentrations, clearly above our established reference ranges, their levels were considerably lower than those observed in HT1 patients at the time of diagnosis.

**TABLE 2:** Concentrations of succinylacetone observed in urine and plasma specimens from individuals with tyrosinemia type 1 or maleylacetoacetate isomerase deficiency.

Source of sample	Diagnosis	Age at sample collection	Plasma SA nmol/L	Urinary SA μmol/mol creatinine
			(Reference < 24)	
				(Reference < 34)
Infant 1	HT1	1 day	93 405^a^	582 492^a^
Infant 2	HT1	10 days	22 132^a^	100 649^a^
Infant 3	HT1	19 days	37 937^a^	337 866^a^
Infant 4	HT1	11 days	31 912^a^	199 186^a^
Infant 5	HT1	1 day	38 896^a^	299 618^a^
Infant 6	MAAID	20 days	1555^b^	1181^b^
Infant 7	MAAID	31 days	384^b^	593^b^
Infant 8	MAAID	21 days	2230^b^	6357^b^
Infant 9	MAAID	71 days	145^b^	478^b^
Infant 10	MAAID	15 days	976^b^	1003^b^
Infant 11	MAAID	40 days	129^b^	191^b^

Abbreviations: SA, Succinylacetone; HT1, Tyrosinemia type 1; MAAID, Maleylacetoacetate isomerase deficiency.

^a^ First specimen received for diagnostic testing; before initiation of treatment for HT1.

^b^ First specimen received for diagnostic testing, following a positive newborn screen.

This method is also applicable to analysis of SA in amniotic fluid (AF), as was described using our previous GC–MS method [13]. We have revised the reference values (now < 3 nmol/L) and confirmed that there is no significant matrix effect with the present GC–MS/MS method. Biochemical prenatal testing for HT1 is occasionally requested. A single abnormal result has been observed, with SA in AF at 1327 nmol/L, and diagnosis subsequently confirmed by molecular analysis of *FAH*.

## 4 Discussion

The method presented here represents a significant enhancement of our original GC–MS method [13]. Now, using GC–MS/MS gives greater selectivity, resulting in less interference of co‐eluting compounds and matrix components, better signal‐to‐noise, easier and more reliable identification of SA using MRM, and better accuracy, especially at low concentrations. The increased sensitivity of the instrument permitted modification of several parameters which reduce sample preparation time and the quantity of material injected into the analyzer, while providing nanomolar level quantitation and improved instrument robustness with less frequent maintenance.

This SA assay is in frequent use in our laboratory, most often for the lifelong monitoring of a large cohort of HT1 patients treated with NTBC [12]. It also constitutes a key step in the process of confirmation or refutation of HT1 diagnosis in “screen positive” infants referred by NBS programs [4, 5] and is sometimes requested to “rule out tyrosinemia” in symptomatic patients for whom the prior suspicion of HT1 is relatively low and the differential diagnosis wide. Given these contexts, an assay calibration range from 0 to 200 nmol/L was chosen to ensure accurate quantitation of even mildly increased SA concentrations and conversely to allow confident reporting of normal results. In our experience, most samples from HT1 patients on long‐term treatment show normal or only mildly elevated SA levels in plasma and urine. Very few samples obtained in this context have concentrations above 200 nmol/L in plasma or above 200 μmol/mol creatinine in urine; an observation which confirms that the choice of calibration range was appropriate. SA results above 200 nmol/L (plasma) or 200 μmol/mol creatinine (urine) prompt rapid communication with the treating physician, and typically are found to reflect an episode of decompensation and/or lack of adherence to NTBC treatment.

In practice, the analytical measurement range extends well above the calibration curve (Table 1), but still is likely to be exceeded in the context of a new diagnosis of an HT1 patient. We therefore use pre‐defined protocols, incorporating a series of appropriate sample dilutions and analysis of these in parallel with analysis of the undiluted specimen in situations where there is reason for strong prior suspicion of HT1.

Challenges and limitations in the accurate analysis of SA in urine, plasma and/or bloodspots, and in result interpretation, have been discussed elsewhere and still pose difficulties in various contexts of screening, diagnosis and monitoring [3, 4, 5, 11, 14, 15, 19, 20, 21, 22, 23, 24]. It is widely accepted that monitoring of HT1 patients on NTBC therapy must include measurement of SA, a surrogate parameter of toxicity, to ensure adequate treatment and optimal outcomes. Clinical guidelines recommend achieving concentrations “below detectable limits” [4, 5], yet limits of detection and quantitation vary widely between laboratories and normal SA levels are well below the LOQs of most laboratories [11, 24]. Since the goal is to maintain SA concentrations within the normal range [5, 11], the sensitivity of this GC–MS/MS method offers a significant advantage. The use of a method which is fully quantitative, highly sensitive and specific also minimizes the likelihood of missing a diagnosis of HT1 due to false negative SA results; in contrast to the greater risk inherent in relying on traditional methods such as qualitative urinary organic acid profile analysis by GC–MS [19].

We have not investigated the application of this GC–MS/MS method to bloodspots, as our laboratory does not perform NBS and because the use of urine and plasma for diagnosis and monitoring is well established in the clinical protocols of the Québec NTBC Study Group [10, 12]. However, the use of bloodspots has certain logistical advantages, particularly a greater stability of SA during specimen storage at room temperature or transport at ambient temperature; therefore, this may be preferred in some other settings, such as clinics offering home monitoring [11]. The sensitivity and specificity of GC–MS/MS could also facilitate assay of bloodspot SA for diagnosis and monitoring, which might be of interest to some biochemical genetics laboratories that are currently adopting GC–MS/MS technology for assay of other metabolites.

Hypersuccinylacetonemia can no longer be considered pathognomonic for HT1 [14, 15], but SA levels are usually lower in MAAI deficiency (Table 2) [14, 15, 25, 26]. Availability of a sensitive assay has helped identify and characterize this condition, which so far appears to be benign and not require treatment with NTBC but which provides an explanation for some “NBS false‐positive” cases. This GC–MS/MS assay is also proving useful in research using model systems to explore pathogenesis and novel treatment approaches for HT1 [27].

To the best of our knowledge, this is the first report of a stable isotope dilution GC–MS/MS method for quantitation of SA. This assay is well suited for diagnosis and monitoring of HT1 and for additional clinical and research applications.

## Author Contributions

Denis Cyr performed the development and validation of the described method, and wrote the first draft of the manuscript. Paula J. Waters contributed to the conception of the study and literature review, and performed critical review and editing of the draft manuscript. Bruno Maranda performed critical review and editing of the draft manuscript. All authors reviewed the final manuscript and gave approval for submission.

## Funding

The authors have nothing to report.

## Disclosure

Informed Consent and Animal Rights: This article does not contain any studies with human or animal subjects performed by any of the authors. All specimens analyzed were originally submitted for clinical laboratory testing. This article does not contain information identifying any individuals, nor does it contain clinical descriptions of individual patients.

## Ethics Statement

Ethics approval was not required for this study, which was performed in accordance with standard practices for method development and validation within a clinical diagnostic laboratory.

## Conflicts of Interest

The authors declare no conflicts of interest.

## Data Availability

The data that support the results and conclusions presented in this article are available from the corresponding author upon reasonable request.
